# Bromide of Potassium Externally as a Caustic and Hæmostatic

**Published:** 1878-10

**Authors:** 


BROMIDE OF POTASSIUM EXTERNALLY AS A CAUSTIC AND HÆMOSTATIC.—Drs. Ferraud and N. Guenean de Mussy (according to *Le Moniteur Therapeutique*) have used bromide of potassium with glycerin in many diseases to relieve local spasms, pruritus, etc. In twenty days, an epithelioma of the face was cured by a daily application of the medicine in powder. They have likewise obtained good results in chronic ulcers of the legs, in diseases of the skin, such as chronic eczema, pityriasis and acne, in stomatitis, and in some phagadenic ulcers. In epistaxis, hæmoptysis and menorrhagias, they found it successful after ergot, perchloride of iron and rhatany had failed.

